# Zinc, nickel, and cobalt ions removal from aqueous solution and plating plant wastewater by modified *Aspergillus flavus* biomass: A dataset

**DOI:** 10.1016/j.dib.2017.04.031

**Published:** 2017-05-01

**Authors:** Rauf Foroutan, Hossein Esmaeili, Seyedehmasomeh Derakhshandeh Rishehri, Farzaneh Sadeghzadeh, Seyedehroghayeh Mirahmadi, Malihe Kosarifard, Bahman Ramavandi

**Affiliations:** a Department of Chemical Engineering, Bushehr Branch, Islamic Azad University, Bushehr, Iran; b Faculty of Health and Nutrition, Bushehr University of Medical Sciences, Bushehr, Iran; c Department of Environmental Health Engineering, Faculty of Health and Nutrition, Bushehr University of Medical Sciences, Bushehr, Iran

**Keywords:** Bioadsorption, *Aspergillus flavus*, Kinetic, Isotherm, Heavy metals, Plating plant wastewater

## Abstract

The biomass of *Aspergillus flavus* was modified by calcium chloride to achieve a bioadsorbent for treating nickel, cobalt, and zinc ions from aqueous solutions. The information of pH, bioadsorbent dose, contact time, and temperature effect on the removal efficiency are presented. The data of Freundlich and Langmuir isotherm and pseudo-first-order and pseudo-second-order kinetic models are also depicted. The data showed that the maximum bioadsorption capacity of nickel, cobalt, and zinc ions is 32.26, 31.06 and 27.86 mg/g, respectively. The suitability of the bioadsorbent in heavy metals removal at field condition was tested with a real wastewater sample collected from a plating plant in the final part of this dataset. Based on the findings, the bioadsorbent was shown to be an affordable alternative for the removal of metals in the wastewater.

**Specifications Table**

**Table:** 

Subject area	*Chemical engineering*
More specific subject area	*Environmental biotechnology*
Type of data	*Table, image, figure*
How data was acquired	– *SEM images of the modified biomass of Aspergillus flavus and used one were prepared using a SEM instrument, Hitachi S4160 model.* – *A flame atomic adsorption spectrometry (SpectrAA-10 Plus, Varian Company) was used for metal ions measurement.* – *- A digital pH meter (Metrohm) was applied for pH analyzing.*
Data format	*Analyzed*
Experimental factors	– *Aspergillus flavus was cultured to obtain fresh biomass.* – *The A. flavus biomass was dried and then modified by CaCl*_*2*_ *and NaOH.* – *The effect of bioadsorbent dosage, pH and temperature was acquired.* – *The isotherm and kinetic parameters were revealed.* – *-A plating wastewater was treated using the bioadsobent.*
Experimental features	*Heavy metals ion bioadsoption by A. flavus biomass*
Data source location	*Bushehr University of Medical Sciences, Bushehr, Iran, GPS: 28.9667°N 50.8333°E*
Data accessibility	*Data represented with the article*

**Value of the data**

• By a simple method the biomass of *Aspergillus flavus* could be modified toward bioadsorption of heavy metal ions.
• This data potentially useful for factories like plating plants with heavy metals laden-wastewaters.
• These data may be useful for scientific community with concern about heavy metal pollution and also will be important for recycling the heavy metals of Zn, Co, and Ni from wastewaters.

## 1 Data

**Fig. 1:**
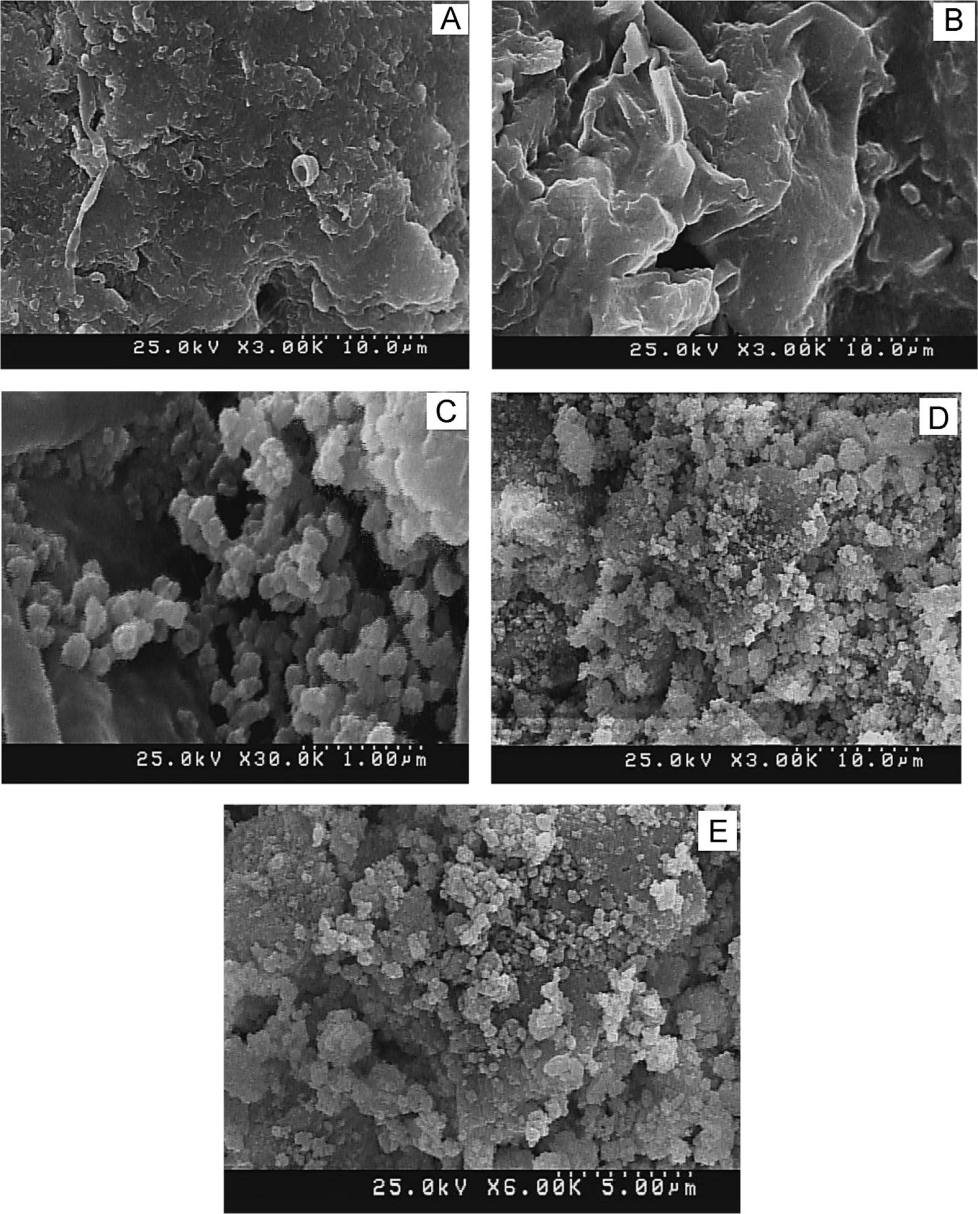
SEM images of *Aspergillus flavus*, A) before modification, B) after modification, C) after bioadsorption of Zn(II), D) after bioadsorption of Co(II), and E) after bioadsorption of Ni(II).

**Fig. 2:**
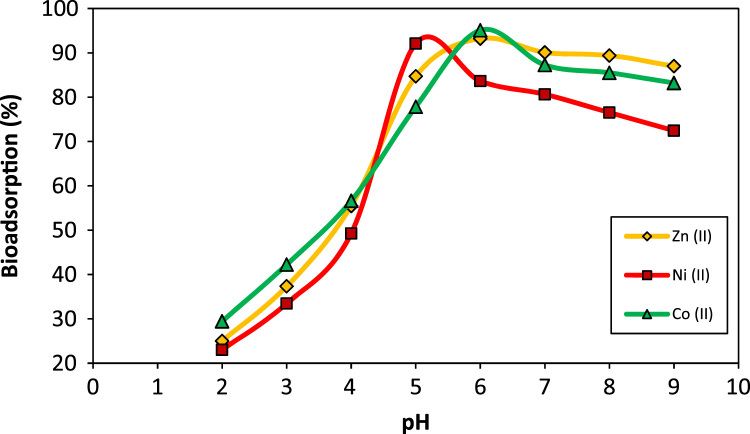
The effect of pH on the bioadsorption of Ni(II), Co(II), and Zn(II) by modified *A. flavus* biomass (metal ions concentration: 50 mg/L, bioadsorbent dose: 4 g/L, contact time: 60 min, temperature: 293.15 K).

**Fig. 3:**
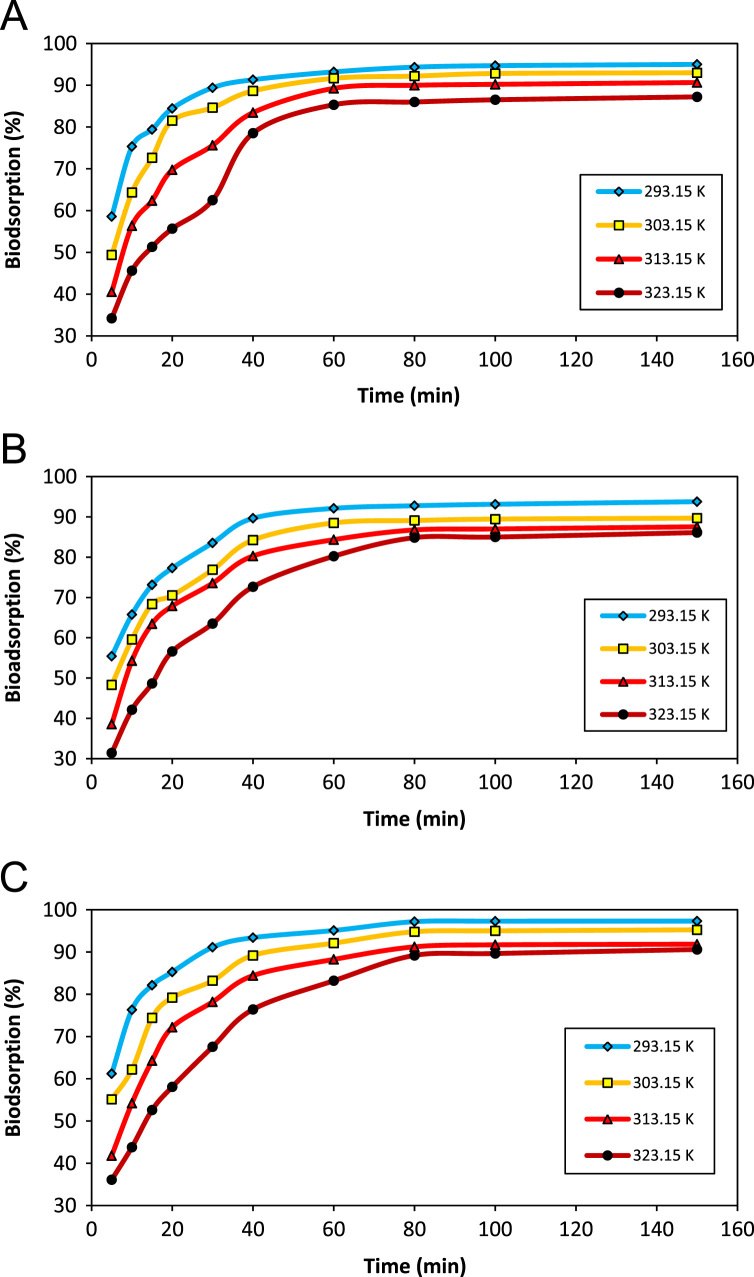
The effect of temperature and contact time on the bioadsorption efficiency of (a) Zn(II), (b) Ni(II), (c) Co(II) by modified *A. flavus* biomass (pH_optimum_, metal concentration: 50 mg/L, bioadsorbent dosage: 4 g/L).

**Fig. 4:**
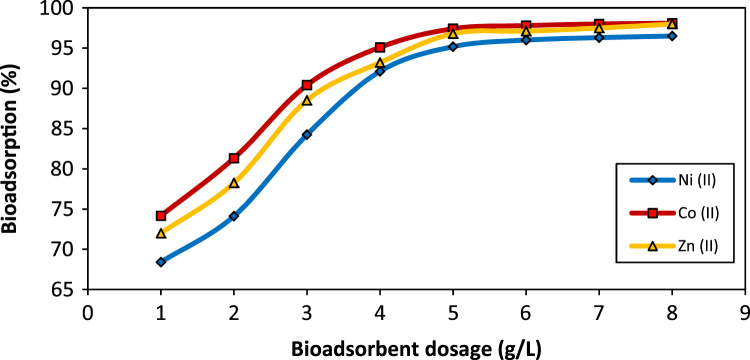
The effect of bioadsorbent dosage on the removal efficacy of Ni(II), Co(II), and Zn(II) by modified *A. flavus* biomass (pH_optimum_, temperature: 293.15 K, time: 60 min, metal concentration: 50 mg/L).

Five scanning electron microscope (SEM) images of *A. flavus* (before modification, after modification, after bioadsorption of Zn, after bioadsorption of Co, and after bioadsorption of Ni) are depicted in Fig. 1. The effect of pH on the bioadsorption of Ni(II), Co(II), and Zn(II) is shown in Fig. 2. Fig. 3 depicts the effect of temperature and contact time on the bioadsorption of Zn(II), Ni(II), and Co(II). The effect of bioadsorbent dosage on the metals removal is also presented in Fig. 4. The isotherm (Langmuir and Freundlich models) and kinetic (pseudo-first-order and pseudo-second-order models) parameters are shown in Table 1, Table 2. The characteristics of plating wastewater before and after treatment with the prepared bioadsorbent are depicted in Table 3.

## 2 Experimental design, materials and methods

### 2.1 Preparation of *Aspergillus flavus* biomass

The locally isolated non-aflatoxin producing *A. flavus* strain was provided from the Microbiology Department of Bushehr University of Medical Sciences, Iran in the form of dry ice. Then, it was cultured in the solid medium contains 40 g/L glucose, 10 g/L peptone, and 15 g/L agar, and kept in 4 °C. For biomass production, the fungi was introduced under sterile conditions to a liquid medium containing: 120 g/L sucrose, 2 g/L NH_4_Cl, 1 g/L KH_2_PO_4_, 0.25 g/L MgSO_4_.7 H_2_O, 0.3 mg/L FeSO_4_, 0.4 g/L ZnSO_4_, 0.15 g/ L MnSO_4_, 0.4 g/L CuSO_4_. Afterward, it was placed in an incubator at a temperature of 28±2 °C and rotation speed of 200 rpm for one week [1]. Upon completion, the biomass of *A. flavus* was collected by filtration of liquid medium and washing several times with double distilled water. The biomass was then placed in the oven at 70 °C for 24 h to be dried, and finally it stored in a closed plastic container at 4 °C for further treatment.

### 2.2 Modification of *A. flavus* biomass

For modification purpose, 2 g achieved biomass was immersed in a solution of 2.0 M calcium chloride for 30 min. Then, 3 g of biomass was refluxed with 100 mL of sodium hydroxide solution for 6 h and after that was filtered by using Whatman filter paper and oven dried at 70 °C for 7 h. Then, separated biomass was washed several times with double distilled water until the pH was reached to 6–7. The rinsed biomass put in autoclave for 30 min at 120 °C, and was dried at a temperature of 60 °C for 24 h [2]. The dried biomass was ground and sieved to obtain the particle with diameter <0.025 mm and used for metal bioadsorption. To ensure the purity of modified biomass, all modification stages were done under aseptic conditions. The density of the bioadsorbent was obtained 1.12 g/cm^3^, which allow the bioadsorbent particles to well-mixed at 200 rpm mixing intensity.

### 2.3 Preparation of metals stock solutions

To prepare the solution containing Zn(II), Ni(II), and Co(II), we used nitrate salts of these metals (*i.e.*, Zn(NO_3_)_2_. 6H_2_O, Ni(NO_3_)_2_. 4H_2_O, and Co(NO_3_) _2_. 6H_2_O, respectively). Stock solution with initial concentration of 1000 mg/L was prepared by solving certain amount of the mentioned salts in double distilled water. To provide the solution heavy metals with concentration of 50 mg/L, the stock solution was diluted by using double distilled water.

### 2.4 Experimental procedure for heavy metals bioadsorption

The bioadsorption tests were carried out in batch mode in 250 mL Erlenmeyer flasks which contain a 100 mL metals solution with concentration of 50 mg/L. To investigate the pH effect on heavy metal ions bioadsorption, the approach of “one variable at the time” was used [3], [4], [5], [6]. To do this, the initial solution pH was set in a range of 2–8. To adjust the solution pH in the desired range, we used hydrochloric acid or sodium hydroxide 0.1 M. After determining the optimum pH in order to evaluate the effect of temperature and contact time on the bioadsorption of heavy metal ions, the bioadsorption process was carried out in the temperature of 293.15–323.15 K, contact time of 5- 150 min, optimal pH, mixing intensity of 200 rpm and bioadsorbent dose of 4 g/L. Similarly, the effect of bioadsorbent dosage (1–8 g/L) on the removal percentage and capacity bioadsorption of heavy metal ions was conducted under optimal conditions.

The removal percentage (R%) and bioadsorption capacity (q_e_) of heavy metal ions were calculated using the following equations [7], [8], [9]:

(1) $$R\%=\frac{C_{0}-C_{f}}{C_{0}}\times 100$$

(2) $$q_{e}=\frac{C_{0}-C_{e}}{W}\times V$$

where, C_i_ and C_f_ is the initial and final concentration of metal ions (mg/L), V is the volume of solution (L), and W is the dry weight of bioadsorbent (g). The optimization tests were repeated three times and average of measurements are stated.

To describe the kinetic behavior of the *A. flavus* biomass to adsorb of nickel, cobalt, and zinc ions from aqueous solutions, the pseudo-first and pseudo-second order models were applied [10], [11]:

(3) $$\mathrm{Pseudo}-\mathrm{first order}\;:\mathrm{Ln}\left(q_{e}-q_{t}\right)=\mathrm{Ln}q_{e}-k_{1}t$$

(4) $$\mathrm{Pseudo}-\mathrm{second order}\;:\frac{t}{q_{t}}=\left(\frac{1}{K_{2}q_{e}^{2}}\right)+\frac{1}{q_{e}}t$$

where, q_e_ (mg/g) is the amount of metal ions sorbed by the bioadsorbent in equilibrium condition per gram of bioadsorbent, q_t_ (mg/g) is the amount of metal ions sorbed per gram of bioadsorbent at any time, and k_1_ (1/min) and k_2_ (mg/g min) are the pseudo-first and pseudo-second order constant, respectively.

To study the isotherm behavior of the adsorption process, Freundlich and Langmuir isotherm models were used [10], [11].

(5) $$\text{Langmuir}\;\text{isotherm}\;\text{model}\;:\frac{1}{q_{e}}=\frac{1}{q_{\max}}+(\frac{1}{bq_{\max}})\frac{1}{C_{e}}$$

where, C_e_ (mg/L) refers to the metal ions concentration at equilibrium, q_e_ (mg/g) is the amount of metal ions adsorbed per gram of adsorbent in equilibrium. q_max_ (mg/g) and b (L/g) are referred to the adsorption capacity and energy respectively, which considered as the constant parameters in the Langmuir model. By measuring the slope and intercept of linear Langmuir Eq., 1/q_e_ was obtained based on 1/C_e_. Another important parameter in Langmuir equation is R_L_ (R_L_=$1/1+bC_{0}$) which represents the characteristics of this isotherm model. If R_L_>1, R_L_=0, R_L_=1, and 0<R_L_<1 the process considered as undesirable, irreversible, linear, and good, respectively [12], [13]. C_0_ (mg/L) refers to the initial concentrations of metal ions in aqueous solution.

(6) $$\text{Freundlich}\;\text{isotherm}\;\text{model}\;:\mathrm{Ln}q_{e}=\mathrm{Ln}K_{f}+\frac{1}{n}\mathrm{Ln}C_{e}$$

where, k_f_ and n are constant parameters of Freundlich model that demonstrate the relationship between bioadsorption capacity and bioadsorption rate. To determine the parameters of 1/n and K_f_, the lnq_e_ draw against lnC_e_. The n parameter represents the interactions between the bioadsorbent and the metal ions, in addition its amount have been reported in the range of 1–10 [14]. If n=1 it is the linear adsorption process, if n>1 it is desirable physical bioadsorption process, and if n<1 it is expressed as chemical bioadsorption process [14].

### 2.5 Sampling and treatment of real wastewater

A bulk of plating wastewater was taken from an active plating plant around Shiraz city, Iran. The sample was collected from equalization tank of the plating wastewater treatment plant. The wastewater properties are listed in Table 3. For treatment purpose, 150 mL wastewater and 4 g/L bioadsorbent was poured in 250 mL flasks and after a 60 min reaction time at 293.15 K temperature and 200 rpm mixing intensity the solutions were passed through 0.45µ-whatman filter. The physic-chemical properties of filtrate were then analyzed (see Table 3). The pH of the wastewater was not corrected to desired amount during the experiment. This test was repeated three times and average values are stated herein.

### 2.6 Measurements

To present surface changes of *A. flavus*, the scanning electron microscope (SEM, Hitachi S4160) was used. In order to capture images of the surface of the bioadsorbent, at first its surface was covered with a thin layer of gold under vacuum condition and then SEM were used [15]. To determine the amount of heavy metal ions remaining in the solution and adjusting the initial pH of solution, we used flame atomic adsorption spectrometry (SpectrAA-10 Plus, Varian Company) and digital pH meter Metrohm, respectively. The density of the bioadsorbent was measured using a laboratory density meter (Mettler Toledo).

## Figures and Tables

**Table 1:** The value of parameters for isotherm models.

Isotherms	Parameters	Metal ion		
		Ni(II)	Co(II)	Zn(II)
Langmuir	q_max_ (mg/g)	32.258	31.056	27.855
	b (l/mg)	0.147	0.292	0.281
	R_L_	0.119	0.064	0.0664
	R^2^	0.9349	0.9419	0.951
Freundlich	n	1.65	1.8	1.79
	K_f_ (mg/g)	4.846	7.168	6.346
	R^2^	0.9127	0.9392	0.9342

**Table 2:** Parameters calculated using pseudo-first-order and pseudo-second-order models for metals bioadsorption by *A. flavus* biomass.

Kinetic model	Parameters	Temperature			
		293.15 K	303.15 K	313.15 K	323.15 K
Pseudo-first-order (Ni)	q_e.cal_	4.79	6.89	6.81	9.09
	k_1_	0.0449	0.0578	0.0486	0.0444
	q_e.exp_	11.721	11.206	10.94	10.65
	R^2^	0.9664	0.9906	0.9887	0.9784
Pseudo-second-order (Ni)	q_e.cal_	12.12	11.709	11.534	11.806
	k_2_	0.019	0.016	0.013	0.0069
	q_e.exp_	11.721	11.206	10.94	10.65
	R^2^	0.9997	0.9994	0.9996	0.9983
Pseudo-first-order (Co)	q_e.cal_	7.121	6.786	9.116	9.714
	k_1_	0.0733	0.0548	0.0591	0.0448
	q_e.exp_	12.165	11.906	11.481	11.325
	R^2^	0.9609	0.9843	0.9863	0.9805
Pseudo-second-order (Co)	q_e.cal_	12.468	12.376	12.136	12.376
	k_2_	0.027	0.0168	0.0121	0.0068
	q_e.exp_	12.165	11.906	11.481	11.325
	R^2^	0.9999	0.9997	0.9995	0.9981
Pseudo-first-order (Zn)	q_e.cal_	3.892	5.603	7.541	9.067
	k_1_	0.048	0.0547	0.0536	0.0499
	q_e.exp_	11.875	11.625	11.3325	10.844
	R^2^	0.9841	0.9872	0.9771	0.9655
Pseudo-second-order (Zn)	q_e.cal_	12.15	12.03	11.99	11.92
	k_2_	0.028	0.02	0.0123	0.0075
	q_e.exp_	11.875	11.625	11.3325	10.844
	R^2^	0.9999	0.9997	0.9992	0.996

**Table 3:** The quality of plating wastewater before and after treatment by the bioadsorbent.

Wastewater quality	Concentration before treatment (mg/L)	Concentration after treatment (mg/L)
Ni(II)	19±4	3±0.7
Cr(VI)	14.5±3	4±0.5
Zn(II)	12±3	ND
Co(II)	8±3	ND
TDS	1045±23	994±9
BOD_5_	245±11	223.4±10
COD	504±25	468.5±14
pH	5.5±0.24	6.8±0.15

ND: Non- detectable.
